# Association of general and abdominal obesity with lung function, FeNO, and blood eosinophils in adult asthmatics: Findings from NHANES 2007–2012

**DOI:** 10.3389/fphys.2023.1019123

**Published:** 2023-02-10

**Authors:** Haoyu Zhang, Zhigang Hu, Sufei Wang, Jiangli Xu, Sijia Li, Xinyu Song

**Affiliations:** ^1^ Department of Respiratory and Critical Care Medicine, The First College of Clinical Medicine Science, China Three Gorges University, Yichang, China; ^2^ Department of Respiratory and Critical Care Medicine, Yichang Central People’s Hospital, Yichang, China; ^3^ Department of Respiratory and Critical Care Medicine, NHC Key Laboratory of Pulmonary Diseases, Union Hospital, Tongji Medical College, Huazhong University of Science and Technology, Wuhan, Hubei, China

**Keywords:** general and abdominal obesity, body mass index, waist circumference, asthma, lung function, FeNO

## Abstract

**Purpose:** Obesity is considered a risk factor for asthma exacerbation. However, limited studies have focused on the association of different levels of weight clusters with asthma. As such, we study the associations between different weight clusters with FeNO, blood eosinophils, and lung function among adult asthmatics.

**Methods:** Data from 789 participants aged 20 years or older in the National Health and Nutrition Examination Survey 2007–2012 were analyzed. Body mass index (BMI) and waist circumference (WC) were used to determine the weight status. The study population was divided into five groups, including normal weight and low WC (153), normal weight and high WC (43), overweight and high WC (67), overweight and abdominal obesity (128), and general and abdominal obesity (398). A Multivariate linear regression model was used to evaluate the abovementioned associations after adjusting for potential confounding factors.

**Results:** The adjusted models showed that general and abdominal obesity cluster (adjusted β = −0.63, 95% confidence interval (CI): −1.08, −0.17 *p* < 0.01), and the normal weight with high WC cluster (adjusted β = −0.96, 95% CI: −1.74, −0.19 *p* < 0.05) were associated with lower levels of blood eosinophils percentage than normal weight and low WC cluster. A similar tendency was shown in the levels of FeNO, but the differences were not significant (*p* > 0.05). Furthermore, abdominal obesity clusters were significantly associated with lower FVC, FVC% predicted, and FEV_1_ measures than normal weight and low WC cluster, especially those individuals with general and abdominal obesity cluster. No association was found between different weight clusters and FEV_1_/FVCF ratio. The two other weight clusters did not show the association with any of the lung function measures.

**Conclusion:** General and abdominal obesity were associated with lung function impairment and a significant reduction of FeNO and blood eosinophil percentage. This study emphasized the importance of concurrent determination of BMI and WC in asthma clinical practice.

## Introduction

Asthma is a heterogeneous disease that is linked to persistent airway inflammation and hyperresponsiveness (Global Initiative for Asthma, 2020). Obesity is a significant risk factor for asthma (Peters et al., 2018). Currently, obesity is now recognised as a significant public health issue in developed countries, because the prevalence of obesity has increased rapidly (Parikh et al., 2007). Asthma phenotypes with different underlying disease processes have been identified, mainly including asthma with obesity, asthma with persistent airway limitation, allergic asthma, non-allergic asthma, and adult-onset asthma (Global Initiative for Asthma, 2020). Asthma with obesity decreases the response to anti-inflammatory therapy and usually encompasses a gambit of a poorly controlled asthma phenotype. Therefore, the effect of asthma with obesity phenotypes needs to be determined for the improvement of treatment modalities.

Body mass index (BMI) and waist circumference (WC) are the most prevalent methods for the measurement of general and abdominal obesity. However, BMI is frequently questioned as a marker of obesity, because it may not precisely provide information about body fat distribution (Hafe et al., 2004). Many studies have reported different associations of general and abdominal obesity with many diseases, including coronary heart disease (Sharma et al., 2016), hypertension (Nurdiantami et al., 2018), type 2 diabetes mellitus (Zhou et al., 2021), and frailty status (Afonso et al., 2021). Several studies have only identified separate effects of general obesity as measured by BMI and abdominal obesity as measured by WC on asthmatics (Goudarzi et al., 2018; Jesus et al., 2018). Fractional exhaled nitric oxide (FeNO) and blood eosinophils are generally considered a marker for type-2 airway inflammation with asthmatics (Bjermer et al., 2014). Some asthma phenotypes are not accompanied by detectable inflammation, including asthma with obesity phenotypes, which do not necessarily involve classical TH2-dependent inflammatory processes (Leiria et al., 2015). Several studies have found lower FeNO and the percentage of sputum eosinophil counted in asthma with obesity phenotypes (Komakula et al., 2007; Lessard et al., 2008). Low levels of FeNO and blood eosinophils are different from obtaining clinical benefits from inhaled corticosteroids treatment (Price et al., 2018). Obesity can induce a change in respiratory physiology, thus impairing lung function (Jesus et al., 2018; Svartengren et al., 2020).

To date, no study has assessed the associations between the combination of general and abdominal obesity and asthma. Accordingly, in the present study, we used simple anthropometric measures of BMI combined with WC to determine the associations of different weight clusters with FeNO, blood eosinophils, and lung function among adult asthmatics.

## Materials and methods

### Study design and population

The National Health and Nutrition Examination Survey (NHANES) is a cross-sectional nationally representative sample conducted by the Centers for Disease Control and Prevention’s National Center for Health Statistics that provides open data on the health and nutritional status of the non-institutionalized civilian resident population of the United States. The NHANES database collected the information through questionnaires and health examinations. In addition, professional interviewers and transportable testing facilities were used to ensure quality control. (https://www.cdc.gov/nchs/nhanes/index.htm).

**FIGURE 1:**
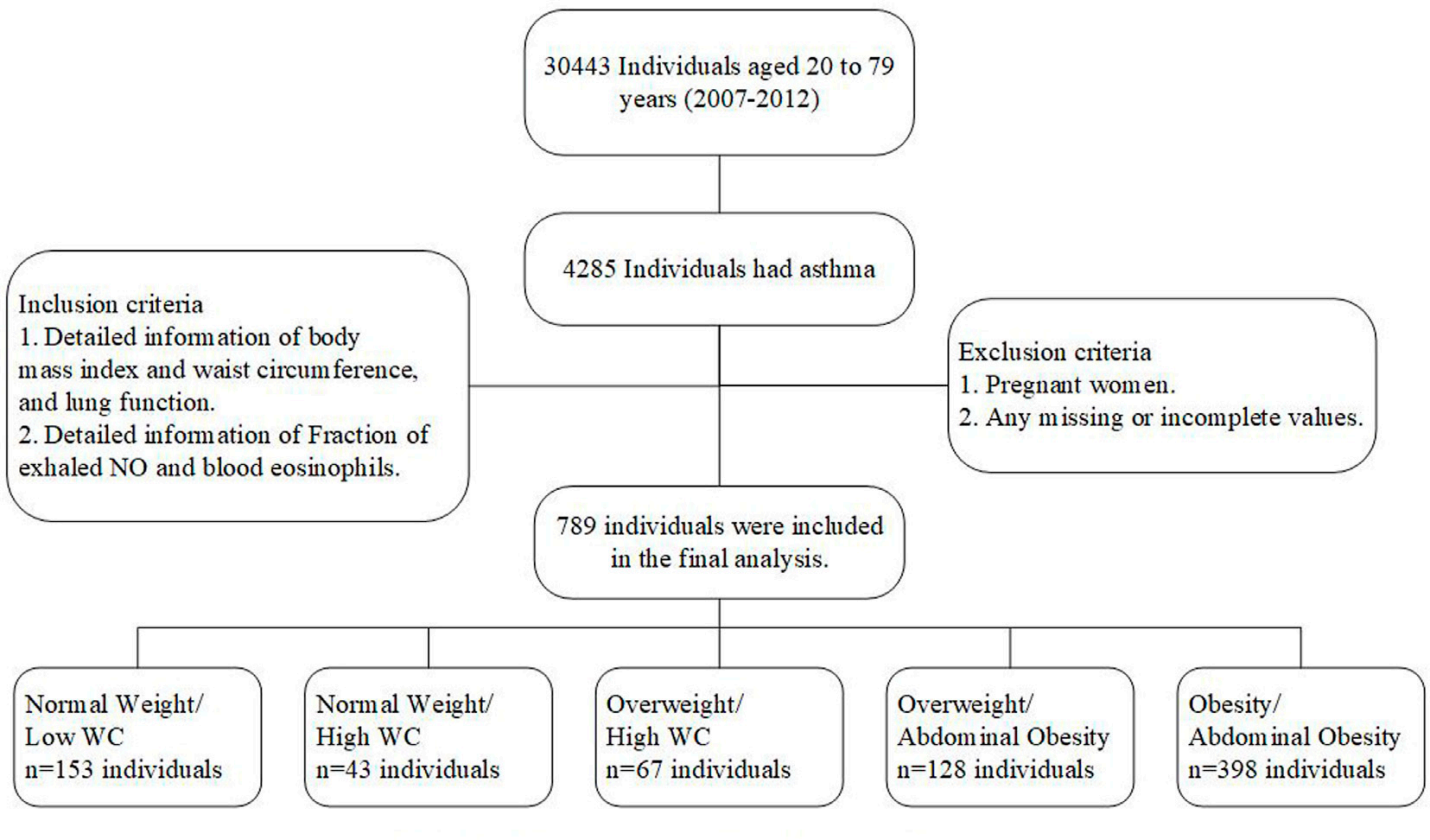
Flow chart of included population.

In this study, an analysis was conducted using the data from three NHANES surveys that included FeNO measurements and spirometry tests: 2007–2008, 2009–2010, and 2011–2012. Asthma was defined as an affirmative response to two questions: First, “Has a doctor ever told you that you had asthma?” Second, “Do you still have asthma?” Asthmatics were assessed and in those who reported asthma, asthmatic exacerbation and asthmatic medical use were specifically assessed by individual questions. First, “Had asthma attack in the past year?”. Second, “Does a doctor prescribe medication for asthma?”. Inclusion criteria were included: First, “Detailed information of body mass index and waist circumference, and lung function”. Second, “Detailed information of Fraction of exhaled NO and blood eosinophils”. Exclusion criteria were included: First, “Pregnant women”. Second, “Any missing or incomplete values”. Finally, 789 participants with asthma aged 20–80 years were selected from NHANES 2007–2012. Detailed data about asthmatics were shown in Figure 1.

The National Center for Health Statistics Research Ethics Review Board approved the protocols (ERB protocol number #2006–2007 and #2011–2017) and the participants provided written informed consent. This study was approved by an institutional review Board at China, Three Gorges University (2021–151–01).

### Interviews and measurements

Weight, height, and WC measures were used to assess obesity in all NHANES participants who attended health screening tests. WC was measured by trained professionals. BMI was calculated as weight to height squared (kg/m^2^). Standard BMI was defined following the World Health Organization (WHO) classification: (normal weight, 18.5–24.9 kg/m^2^; overweight, 25–29.9 kg/m^2^; obesity, ≥ 30 kg/m^2^) (World Health Organization, 2000). WC was categorized into the following categories: low WC (men, WC ≤ 94 cm; women, WC ≤ 80 cm); high WC (men, WC = 95–102 cm; women, WC = 81–88 cm); and abdominal obesity (men, WC > 102 cm; women, WC > 88 cm) (World Health Organization, 2011). On the basis of the combination of BMI and WC category in asthmatics, five weight clusters were obtained: 1) normal weight/low WC, 2) normal weight/high WC, 3) overweight/high WC, 4) overweight/abdominal obesity, 5) obesity/abdominal obesity. Expected, in this study, general obesity without abdominal obesity cluster and overweight with low WC cluster were rare in adult asthmatics. Therefore, these individuals were not considered.

Meanwhile, this research focused on FeNO, blood eosinophils, and spirometry. First, spirometry was performed in accordance with the recommendations from the American Thoracic Society (Miller et al., 2005). An Ohio 822/827 dry-rolling seal volume spirometer was used to quantify spirometry. The following measures were used to evaluate lung function: forced expiratory volume in the first second (FEV_1_), forced vital capacity (FVC), and FEV_1_/FVC ratio. FEV_1_ and FVC were also calculated as the percentage of predicted values for each participant in accordance with Hankinson’s predicted value equation (Hankinson et al., 1999). An electrochemical sensor was used to determine the FeNO level by using a handheld device (NIOXMINO, Aerocrine, Stockholm, Sweden). All participants underwent two FeNO tests that were repeatable, and the mean value was determined. Venous blood count was assessed using Beckman Coulter’s (Beckman Coulter, Fullerton, CA) method of counting and sizing. The number and proportion of blood eosinophils were determined to assess the systemic inflammation of patients with asthma. The detailed inspection procedures are publicly available (https://www.cdc.gov/nchs/nhanes/index.htm).

### Covariates

Considering potential confounders associated with obesity and asthma, the following factors were controlled in accordance with existing literature (Mogensen et al., 2018; Myung et al., 2018). Therefore, we included information on sociodemographic characteristics (gender, age, race, marital status, education level), lifestyle (smoking status, physical activity, sleep disorder, and insomnia), comorbid conditions (diabetes, arthritis, chronic heart diseases, lung emphysema, and chronic bronchitis), and other covariates (asthmatic exacerbation, and medical use) were collected at baseline through self-reports. Three sets of models were applied with an increasing degree of adjustment. Model 1 was adjusted for age, gender, race, education level, and marital status. Model 2 carried the components of model 1, plus smoking status, physical activity, insomnia, and sleep disorder. Model 3 carried the components of model 2 plus diabetes, arthritis, chronic heart diseases, lung emphysema, chronic bronchitis, asthmatic exacerbation, and medical use.

### Statistical analysis

Empower(R) http://www.empowerstats.net/analysis/ and R software, version 3.1.2 (www.r-project.org) was utilized to portray and evaluate the differences in the demographic and clinical characteristics among the five weight clusters. Using the proposed weighting methodology with the decreased risk of oversampling and non-response bias usefully ensured that estimates are representative of the general US population (National Center for Health Statistics and Centers for Disease Control and Prevention, 2005).

Data were presented as numbers and percentage (%) in parenthesis for categorical variables and estimated mean ± standard errors (SE) for continuous variables. The Rao-Scott χ2 test for categorical variables and analysis of variance (ANOVA) for continuous variables were used to compare baseline characteristics (Rao and Scott, 1984). Multivariate linear regression analyses were also performed to determine the associations between different weight clusters and lung function, FeNO levels, and blood eosinophils among asthmatics. In the statistical studies, regression coefficient (β) and 95% confidence interval (CI) were utilized to explain the differences, and a two-tailed *p* < 0.05 was considered statistically significant.

## Results

### Baseline characteristics of participants

Overall, 789 participants selected from the available NHANES were included in the final analysis. The number of participants who were normal weight and low WC, normal weight and high WC, overweight and high WC, overweight and abdominal obesity, and general and abdominal obesity were 153, 43, 67, 128, and 398 participants, respectively. In total, 50.4% of the participants had general obesity and abdominal obesity. The mean age of the participants was 45.4 (±16.1) years and most asthmatics were women (62.2%). Compared with participants who were normal weight and low WC, those with general and abdominal obesity were more inclined to be female, older, and have more concurrent comorbidities. As Table 1 shown, different weight clusters were associated with the different covariates as follows: gender, age, race, marital status, sleep situation, comorbid conditions (diabetes, arthritis, lung emphysema), and lung function (*p* < 0.05). More detailed information on the different weight clusters were presented in Table 1.

**TABLE 1:** Baseline characteristic of the study clusters.

	Normal weight		Overweight		Obesity	*p*-value
Variable	Low WC	High WC	High WC	Abdominal obesity	Abdominal obesity	
N (%)	153 (19.39)	43 (5.45)	67 (8.49)	128 (16.22)	398 (50.44)	
Gender, N (%)						<0.01
Male	93 (60.59)	7 (17.17)	43 (64.88)	31 (24.52)	129 (32.32)	
Female	60 (39.41)	36 (82.83)	24 (35.12)	97 (75.48)	269 (67.68)	
Age, N (%)						<0.01
20–39 years	102 (66.65)	11 (26.50)	22 (33.34)	38 (29.75)	140 (35.30)	
40–59 years	39 (25.75)	25 (57.74)	33 (48.44)	50 (39.08)	160 (40.19)	
≥ 60 years	12 (7.60)	7 (15.75)	12 (18.23)	40 (31.17)	98 (24.51)	
Race, N (%)						<0.01
Mexican	6 (3.70)	1 (2.36)	3 (4.40)	3 (2.22)	20 (5.06)	
Other Hispanic	7 (4.85)	2 (3.69)	4 (5.96)	6 (5.13)	19 (4.67)	
White, Non-Hispanic	113 (74.00)	36 (84.60)	47 (69.79)	108 (84.13)	274 (68.81)	
Black, Non-Hispanic	18 (11.43)	1 (3.32)	9 (13.03)	7 (5.53)	70 (17.59)	
Other	9 (6.02)	3 (6.02)	4 (6.81)	4 (3.00)	15 (3.87)	
Education, N (%)						0.34
< High school	20 (13.34)	5 (12.68)	11 (16.89)	17 (13.47)	72 (17.99)	
High school	31 (20.19)	10 (22.19)	17 (25.86)	19 (14.53)	103 (25.83)	
> High school	102 (66.48)	28 (65.14)	39 (57.25)	92 (72.00)	223 (56.18)	
Marital status, N (%)						<0.01
Married	64 (41.80)	34 (77.67)	45 (67.49)	87 (68.25)	236 (59.33)	
Widow	4 (2.28)	0 0)	1 (0.34)	6 (4.27)	17 (4.32)	
Divorced/Separated	14 (9.37)	5 (12.30)	5 (8.28)	16 (12.66)	79 (19.88)	
Never married	71 (46.55)	4 (10.04)	16 (23.89)	19 (14.82)	66 (16.47)	
Smoking, N (%)						0.16
Yes	59 (38.58)	12 (28.06)	19 (28.82)	38 (29.46)	96 (24.20)	
No	94 (61.42)	31 (71.94)	48 (71.18)	90 (70.54)	302 (75.80)	
Vigorous activity, N (%)						0.35
Yes	40 (26.40)	13 (29.87)	10 (14.51)	27 (21.36)	79 (19.94)	
No	113 (73.60)	30 (70.13)	57 (85.49)	101 (78.64)	319 (80.06)	
Asthmatic exacerbation, N (%)						0.76
Yes	75 (49.20)	17 (40.37)	30 (45.14)	64 (50.33)	205 (51.40)	
No	78 (50.80)	26 (59.63)	37 (54.86)	64 (49.67)	193 (48.60)	
Asthmatic medical use						0.76
Yes	91 (59.39)	30 (69.08)	46 (68.58)	77 (60.48)	253 (63.47)	
No	62 (40.61)	13 (30.92)	21 (31.42)	51 (39.52)	145 (36.53)	
Insomnia, N (%)						0.01
Yes	39 (25.45)	14 (32.58)	27 (39.71)	58 (44.92)	183 (46.07)	
No	114 (74.55)	29 (67.42)	40 (60.29)	70 (55.08)	215 (53.93)	
Sleep disorder, N (%)						<0.01
Yes	9 (5.91)	6 (13.58)	9 (13.57)	16 (12.88)	90 (22.52)	
No	144 (94.09)	37 (86.42)	58 (86.43)	112 (87.12)	308 (77.48)	
Diabetes, N (%)						<0.01
Yes	2 (1.54)	1 (0.92)	4 (6.51)	9 (7.11)	81 (20.45)	
No	151 (98.46)	42 (99.08)	63 (93.49)	119 (92.89)	317 (79.55)	
Arthritis, N (%)						<0.01
Yes	24 (15.47)	10 (24.14)	11 (16.27)	47 (36.37)	183 (46.02)	
No	129 (84.53)	33 (75.86)	56 (83.73)	81 (63.63)	215 (53.98)	
Chronic heart diseases, N (%)						0.13
Yes	2 (1.11)	3 (7.11)	1 (0.70)	2 (1.21)	15 (3.66)	
No	151 (98.89)	40 (92.89)	66 (99.30)	126 (98.79)	383 (96.34)	
Lung emphysema, N (%)						<0.01
Yes	4 (2.66)	1 (2.64)	1 (0.73)	13 (10.12)	24 (5.92)	
No	149 (97.34)	42 (97.36)	66 (99.27)	115 (89.88)	374 (94.08)	
Chronic bronchitis, N (%)						0.15
Yes	20 (13.15)	12 (28.50)	14 (21.12)	37 (29.29)	99 (24.87)	
No	133 (86.85)	31 (71.50)	53 (78.88)	91 (70.71)	299 (75.13)	
Body mass index (kg/m^2^)	23.1 ± 0.3	23.4 ± 0.2	27.1 ± 0.2	27.8 ± 0.1	37.3 ± 0.4	<0.01
Waist circumference (cm)	82.5 ± 0.7	86.6 ± 1.1	93.9 ± 1.0	99.1 ± 0.8	116.7 ± 1.1	<0.01
Blood eosinophil percentage (%)	3.55 ± 0.18	3.1 ± 0.65	3.95 ± 0.52	3.39 ± 0.22	3.1 ± 0.13	0.10
Blood eosinophil count (10^9^ L^−1^)	0.23 ± 0.02	0.22 ± 0.06	0.28 ± 0.04	0.23 ± 0.01	0.24 ± 0.01	0.59
FeNO (ppb)	24.06 ± 2.28	19.98 ± 5.23	23.59 ± 2.72	19.98 ± 1.99	22.55 ± 1.84	0.69
FVC (mL)	4,397.46 ± 89.8	3,867.29 ± 255.82	4,190.75 ± 173.38	3,785.05 ± 112.41	3,557.76 ± 72.32	<0.01
FEV_1_ (mL)	3,279.21 ± 102.71	2,833.66 ± 211.29	3,058.12 ± 142.6	2,753.71 ± 93.98	2,666.34 ± 51.53	<0.01
FEV_1_/FVC	0.74 ± 0.01	0.72 ± 0.02	0.73 ± 0.02	0.72 ± 0.01	0.75 ± 0.01	0.09
FVC% predicted	91.2 ± 1.2	87.9 ± 4.4	93.3 ± 1.9	85.9 ± 1.5	83.1 ± 0.8	<0.01
FEV_1_% predicted	83.4 ± 1.8	80.9 ± 4.8	86.1 ± 2.3	79.3 ± 1.6	79.0 ± 1.0	0.02
FEV_1_/FVC% predicted	0.91 ± 0.02	0.91 ± 0.02	0.92 ± 0.02	0.92 ± 0.01	0.95 ± 0.01	0.14

All estimates accounted for complex survey design. Data are presented as number with weighted percentage (%) or weighted mean ± standard error.

Normal weight, overweight, and obese were defined using standard BMI, cutoffs (normal BMI, 18.5–24.9 kg/m^2^; overweight, 25–29.9 kg/m^2^; obese, ≥30 kg/m^2^); Waist circumference categorized as low WC (≤80 cm for women and ≤94 cm for men), high WC (81–88 cm for women and 95–102 cm for men) and abdominal obesity (> 88 cm for women and > 102 cm for men.

BMI, body mass index; WC, waist circumference; FEV_1_, forced expiratory volume in 1s; FVC, forced vital capacity; FeNO, fraction of exhaled NO.

### Association of different weight clusters with the level of FeNO and blood eosinophils in adult asthmatics

The associations of different weight clusters with FeNO and blood eosinophils were summarized in Table 2. The results showed that normal weight with high WC cluster (adjusted β = −0.96, 95%CI: −1.74, −0.19 *p* < 0.05, in model 3), and general and abdominal obesity cluster (adjusted β = −0.63, 95% CI: −1.08, −0.17 *p* < 0.01, in model 3) were associated with lower levels of blood eosinophils percentage than normal weight and low WC cluster. However, adjusted β indicated no significant difference in the levels of blood eosinophils count (all *p* > 0.05). Meanwhile, after adjusting for potential confounding factors, normal weight with high WC cluster (adjusted β = −6.06, 95%CI: −12.93, 0.81, in model 3), and general and abdominal obesity cluster (adjusted β= −3.44, 95%CI: −7.46, 0.59, in model 3) were found to have lower levels of FeNO than normal weight and low WC cluster. Unfortunately, these differences were not significant (*p* > 0.05) (see Table 2).

**TABLE 2:** Association of different weight clusters with the level of FeNO and blood eosinophil count in adult asthmatics.

	Normal weight		Overweight		Obesity
	low WC	High WC	High WC	Abdominal obesity	Abdominal obesity
Blood eosinophil percentage					
Model 1	Reference	−0.92(−1.70, −0.14)^*^	−0.11 (−0.77, 0.56)	−0.50 (−1.06,0.05)	−0.64(−1.08, −0.20)^**^
Model 2	Reference	−0.92(−1.70, −0.14)^*^	−0.08 (−0.75, 0.58)	−0.46 (−1.02,0.09)	−0.58(-1.03, −0.13)^*^
Model 3	Reference	−0.96(−1.74, −0.19)^*^	−0.10 (−0.76, 0.56)	−0.44 (−1.00, 0.11)	−0.63(−1.08, −0.17)^**^
**Blood eosinophil counts**					
Model 1	Reference	−0.04 (−0.10, 0.02)	0.03 (−0.02, 0.08)	0.00 (−0.04, 0.04)	0.01 (−0.03, 0.04)
Model 2	Reference	−0.04 (−0.10, 0.02)	0.04 (−0.01, 0.09)	0.00 (−0.04, 0.05)	0.02 (−0.02, 0.05)
Model 3	Reference	−0.04 (−0.10, 0.02)	0.04 (−0.01, 0.09)	0.01 (−0.04, 0.05)	0.01 (−0.02, 0.05)
**FeNO**					
Model 1	Reference	−4.83 (−1.85, 2.18)	−1.78 (−7.75, 4.20)	−3.47 (−8.44, 1.51)	−3.07 (−7.03, 0.89)
Model 2	Reference	−5.68 (−12.57, 1.21)	−2.09 (−7.95, 3.78)	−3.40 (−8.29, 1.49)	−3.76 (−7.72, 0.21)
Model 3	Reference	−6.06 (−12.93, 0.81)	−2.38 (−8.22, 3.47)	−3.20 (−8.09, 1.68)	−3.44 (−7.46, 0.59)

Regression coefficient β (95% confidence interval).

^*^ *p* < 0.05; ^**^ *p* < 0.01.

Model 1: adjusted for age, sex, race, education, marital status.

Model 2: model 1 plus adjusted for smoking, vigorous activity, insomnia, sleep disorder.

Model 3: model 2 plus diabetes, arthritis, chronic heart diseases, lung emphysema, chronic bronchitis, asthmatic exacerbation and medical use.

BMI, body mass index; WC, waist circumference; FeNO, fraction of exhaled NO.

### Association of different weight clusters with lung function in adult asthmatics

The associations of different weight clusters with lung function were summarized in Table 3. The results showed that general and abdominal obesity cluster (adjusted β = −237.38, 95%CI: −388.62, −86.14 *p* < 0.01, in model 3), and overweight and abdominal obesity cluster (adjusted β = −129.17, 95%CI: −313.19, 54.85 *p* < 0.01, in model 3) had significantly lower FVC than normal weight and low WC cluster. Meanwhile, general and abdominal obesity cluster (adjusted β = −3.62, 95%CI: −6.19, −1.06 *p* < 0.01, in model 3), and overweight and abdominal obesity cluster (adjusted β = −1.12, 95%CI: −4.24, 2.01 *p* < 0.01, in model 3) were found to be significantly inversely associated with FVC%. Similar to the findings on FVC and FVC%, general and abdominal obesity cluster (adjusted β = −181.86, 95%CI: −320.95, −42.77 *p* < 0.05, in model 3) and overweight and abdominal obesity cluster (adjusted β = −190.19, −359.43, −20.95 *p* < 0.05, in model 3) had lower FEV_1_ than normal weight and low WC cluster. However, adjusted β indicated no associations between the two other weight clusters and any of the lung function measures than normal weight and low WC cluster (see Table 3). Different weight clusters had no significant effect on FEV_1_/FVC ratio.

**TABLE 3:** Association of different weight clusters with lung function in adult asthmatics.

	Normal weight		Overweight		Obesity
	low WC	High WC	High WC	Abdominal obesity	Abdominal obesity
FVC					
Model 1	Reference	−92.86 (−374.98, 189.26)	−213.93 (−447.64, 19.78)	−299.78 (−499.01, −100.55)^**^	−502.26 (−659.27, -345.26)^**^
Model 2	Reference	−98.50 (--379.34, 182.34)	−191.82 (−424.66, 41.03)	−281.22 (−479.92, −82.51)^**^	−461.48 (−620.91, -302.05)^**^
Model 3	Reference	−74.25 (−331.61, 183.12)	−124.02 (−337.81, 89.77)	−129.17 (−313.19, 54.85)^**^	−237.38 (−388.62, -86.14)^**^
**FVC%**					
Model 1	Reference	0.20 (−4.29, 4.70)	1.47 (−2.25, 5.20)	−2.47 (−5.64, 0.71)^**^	−6.07 (−8.57, −3.57)^**^
Model 2	Reference	0.08 (−4.40, 4.56)	1.45 (−2.26, 5.17)	−2.36 (−5.53, 0.81)^**^	−5.79 (−8.34, −3.25)^**^
Model 3	Reference	0.39 (−3.98, 4.76)	2.11 (−1.52, 5.74)	−1.12 (−4.24, 2.01)^**^	−3.62 (−6.19, −1.06)^**^
**FEV**_**1**_					
Model 1	Reference	−188.97 (−449.30, 71.35)	−215.15 (−430.81, 0.51)	−337.49 (−521.32, −153.65)^**^	−395.89 (-540.76, −251.01)^**^
Model 2	Reference	−206.46 (−466.30, 53.38)	−208.09 (−423.53, 7.34)	−334.16 (−518.00, −150.31)^**^	−382.14 (−529.64, −234.62)^**^
Model 3	Reference	−178.94 (−415.63, 57.75)	−145.87 (−342.48, 50.75)	−190.19 (−359.43, −20.95)^*^	−181.86 (−320.95, −42.77)^*^
**FEV**_**1**_ **%**					
Model 1	Reference	−1.37 (−6.86, 4.12)	2.26 (−2.28, 6.81)	−2.71 (−6.59, 1.16)	−3.34 (−6.39, −0.28)^*^
Model 2	Reference	−1.93 (−7.40, 3.53)	1.72 (−2.81, 6.25)	−3.10 (−6.97, 0.76)	−3.91 (−7.01, −0.81)^*^
Model 3	Reference	−1.39 (−6.71, 3.93)	2.32 (−2.10, 6.74)	−1.91 (−5.71, 1.90)	−2.10 (−5.23, 1.02)
**FEV**_**1**_ **/FVC**					
Model 1	Reference	−0.04 (−0.07, −0.01)	−0.02 (−0.05, 0.01)	−0.04 (−0.06, −0.01)	−0.01 (−0.02, 0.01)
Model 2	Reference	−0.04 (−0.08, −0.01)	−0.02 (−0.05, 0.01)	−0.04 (−0.06, −0.02)	−0.01 (−0.03, 0.01)
Model 3	Reference	−0.04 (−0.07, −0.01)	−0.02 (−0.05, −0.01)	−0.03 (−0.05, −0.01)	0.00 (−0.02, 0.02)
**FEV**_**1**_ **/FVC%**					
Model 1	Reference	−0.03 (−0.07, 0.01)	−0.00 (−0.03, 0.04)	−0.01 (−0.04, 0.02)	0.03 (0.00, 0.05)
Model 2	Reference	−0.03 (−0.07, 0.01)	−0.00 (−0.04, 0.03)	−0.02 (−0.04, 0.01)	0.02 (−0.01, 0.04)
Model 3	Reference	−0.03 (−0.07, 0.01)	−0.00 (−0.04, 0.03)	−0.01 (−0.04, 0.02)	0.02 (−0.01, 0.04)

Regression coefficient β (95% confidence interval).

^*^ *p* < 0.05; ^**^ *p* < 0.01.

Model 1: adjusted for age, sex, race, education, marital status.

Model 2: model 1 plus adjusted for smoking, vigorous activity, insomnia, sleep disorder.

Model 3: model 2 plus diabetes, arthritis, chronic heart diseases, lung emphysema, chronic bronchitis, asthmatic exacerbation and medical use.

BMI, body mass index; WC, waist circumference; FEV_1_, forced expiratory volume in 1s; FVC, forced vital capacity.

## Discussion

This study showed the associations between different weight clusters and lung function, FeNO, and blood eosinophils in adult asthmatics by analyzing the representative samples from the NHANES. After adjusting possible potential confounding factors, this study observed that the combination of general obesity and abdominal obesity, and normal weight with high WC cluster had a lower level of blood eosinophils percentage in adult asthmatics than normal weight and low WC cluster. Furthermore, asthmatics with abdominal obesity clusters were associated with lung function impairment manifested by the reduction of FVC, FEV_1_, and FVC% measures, especially those individuals with general and abdominal obesity cluster. While no associations were found between two other weight clusters and any of the lung function measures than normal weight and low WC cluster. In addition, we observed racial differences and marital status may affect obesity in asthma in this study. These findings seem to indicate the importance of race/marital status as a risk factor for obesity in asthma. But the relationship between them is still unclear. This study was the first study to simultaneously analyse the effects of various combinations of BMI and WC on airway inflammation and lung function in adult asthmatics.

The previous study discovered the connection of sleep duration with FeNO level, blood eosinophil percentage, and lung function among asthmatics (Hu et al., 2021). The results suggested that abdominal obesity has more effect on the link between sleep duration and asthma than general obesity (Hu et al., 2021). In this study, the combination of BMI and WC into a single obesity phenotypic indicator was used to determine the associations between different weight clusters and asthma.

The association between obesity, as measured by BMI, and FeNO remains debatable. The analysis results from two sample representative populations show that obesity assessed by BMI is negatively associated with FeNO (Barros et al., 2006; Komakula et al., 2007). By contrast, a cross-sectional study analysis involving 10,817 adults showed that obesity assessed by BMI is not linked to FeNO (Singleton et al., 2014). This finding was obtained, because BMI was unable to accurately assess adiposity, distinguish muscle from fat and reflect body fat distribution. Some published studies questioned the use of BMI alone as a general indicator of obesity in obesity-related studies (Daniels et al., 1997; Goyal et al., 2014). WC has been regarded as a better indicator of adiposity than BMI. However, none of the published studies focused on the influence of combining BMI and WC on individuals suffering from asthma. In the present study, adult asthmatics were also observed to have lower FeNO levels in abdominal and general obesity cluster than normal weight and low WC cluster although the results were not statistically significant. The underlying mechanisms related to obesity and the levels of low FeNO remain unclear. Studies on a murine model of obesity-related asthma showed that adiponectin secreted by adipose tissue can alleviate the exacerbation of airway inflammation and increase airway oxidative stress through the AMPK signalling pathway (Zhu et al., 2019), which reduces NO production from endothelial cells. The effect of general and abdominal obesity cluster on the levels of blood eosinophil percentages was also observed. Blood eosinophils are linked to asthma severity (Busse et al., 2013) and have a significantly positive relationship with the percentage of sputum eosinophil count (Zhang et al., 2014). Advance investigation proposed that the percentage of blood eosinophils is the finest indicator of eosinophilic asthma with an area under the curve of 0.907 (Zhang et al., 2014). A small group of obese asthmatics study showed an inverse correlation between sputum eosinophils and high WC or BMI (Lessard et al., 2008). Adiponectin alleviates airway inflammation in obesity-related mice, accounting for the relatively lower eosinophils (Zhu et al., 2019). In addition, asthma in obese individuals is presumed to have a more commonly non-eosinophilic immunopathological mechanism (Scott et al., 2011). Overall, a low level of blood eosinophils may be an indicator of decreased airway inflammation and non-eosinophilic asthma. Results showed that the abdominal and general obesity cluster is associated with a significant reduction in blood eosinophils percentage. Interestingly, normal weight and high WC cluster was associated with the lowest level of blood eosinophils percentage among the five weight clusters. This phenomenon remains to be identified and studied further.

Two studies observed the combined effect of BMI and abdominal obesity on lung function in general populations (Pan et al., 2017; Svartengren et al., 2020). The association of general obesity with an impaired lung function is conditional on the existence of abdominal obesity (Svartengren et al., 2020). Another cohort study included 16,186 Chinese people who were separated into groups based on their adiposity indices. Based on the comparison of the β values of each group, those people with general and abdominal obesity were associated with lower FEV_1_% and FVC% (Pan et al., 2017), but two studies found have no effect on the FEV_1_/FVC ratio. However, in adult asthmatics, no study has shown the combined effect of general obesity and abdominal obesity on lung function. In the present study, a negative association with lung function was mainly found in the abdominal obesity clusters, especially those individuals with general and abdominal obesity cluster. But no associations were found between two other weight clusters and any of the lung function measures than normal weight and low WC cluster. This finding supports the hypothesis that the link between this obesity cluster and impaired lung function can mainly be mediated through abdominal obesity. This phenomenon is attributed to the destruction caused by the mass load of adipose tissue around the abdomen and in the visceral cavity, with the following mechanisms being proposed explaining the link between these weight clusters and the impairment of lung function. Firstly, these weight clusters directly affect the reduction of diaphragmatic movement and chest-wall expansion, which potentially reduce respiratory volumes (Brashier and Salvi, 2013). Secondly, the long-term chronic and persistent pro-inflammatory state contribute to increasing airway wall thickness and vascular congestion of the airway wall, even further damaging lung function (Mafort et al., 2016). Thirdly, the reduction of FeNO levels can cause pulmonary vascular endothelial dysfunction and lead to impaired lung function (Ouedraogo et al., 2007). Thus, the negative association between BMI and lung function (Ochs-Balcom et al., 2006) might be muddled or mediated in part by WC, which appears to be explained by the fact that abdominal obesity has taken the position of overall obesity.

This study has several limitations to be considered. Firstly, it is a cross-sectional study design that did not allow us to conclude a causality relationship between general and abdominal obesity phenotype, lung function, and airway inflammation in asthmatics. Secondly, current asthma diagnosis and treatment were self-reported and may harbor recall bias. Thirdly, other markers of abdominal obesity, such as the waist-to-height ratio or the waist-hip ratio, were studied because of the lack of data or a small sample size. Future research employing alternative measures of abdominal obesity, such as the waist-to-height ratio or the waist-hip ratio, will be required to confirm our findings. Fourth, although adjustments were made for possible confounding factors, we could not control all potential variables and completely rule out residual confounding. Finally, due to the extrapolation of the study population from a prior database, self-reported asthma diagnosis and the exclusion of the pharmacological therapy in the inclusion criteria make this study non-comprehensive. Despite these limitations, our study has some advantages, including the large study population, a wide range of covariates adjusted and simultaneous exploration of the associations between different weight clusters with FeNO, blood eosinophils, and lung function in adult asthmatics. Furthermore, this study not only combined BMI and WC into a single obesity phenotypic indicator to explore the relationship between asthma and anthropometric indicators of physical obesity, but also went into greater detail by considering intermediate WC categories to determine the associations between asthma and various levels of abdominal adiposity.

## Conclusion

Asthmatic patients with general and abdominal obesity were associated with lung function impairment and resulted in lower levels of blood eosinophil percentages and FeNO than those with normal BMI and low WC. Therefore, BMI and WC measurements need to be combined when assessing asthma-related risk in adults. Further studies are warranted to explore the mechanism of normal weight and high WC in affecting airway inflammation.

## Data Availability

The datasets presented in this study can be found in online repositories. The names of the repository/repositories and accession number(s) can be found below: https://www.cdc.gov/nchs/nhanes/index.htm.
